# Detection of Oil Spill in SAR Image Using an Improved DeepLabV3+

**DOI:** 10.3390/s24175460

**Published:** 2024-08-23

**Authors:** Jiahao Zhang, Pengju Yang, Xincheng Ren

**Affiliations:** 1 School of Physics and Electronic Information, Yan’an University, Yan’an 716000, China; zjh2812480971@yau.edu.cn; 2 Key Laboratory for Information Science of Electromagnetic Waves (MoE), Fudan University, Shanghai 200433, China; 3 Shaanxi Key Laboratory of Intelligent Processing for Big Energy Data, Yan’an 716000, China; xchren@yau.edu.cn

**Keywords:** oil spill detection, DeepLabV3+, MobileNetV2, scSE, joint loss function

## Abstract

Oil spill SAR images are characterized by high noise, low contrast, and irregular boundaries, which lead to the problems of overfitting and insufficient capturing of detailed features of the oil spill region in the current method when processing oil spill SAR images. An improved DeepLabV3+ model is proposed to address the above problems. First, the original backbone network Xception is replaced by the lightweight MobileNetV2, which significantly improves the generalization ability of the model while drastically reducing the number of model parameters and effectively addresses the overfitting problem. Further, the spatial and channel Squeeze and Excitation module (scSE) is introduced and the joint loss function of Bce + Dice is adopted to enhance the sensitivity of the model to the detailed parts of the oil spill area, which effectively solves the problem of insufficient capture of the detailed features of the oil spill area. The experimental results show that the mIOU and F1-score of the improved model in an oil spill region in the Gulf of Mexico reach 80.26% and 88.66%, respectively. In an oil spill region in the Persian Gulf, the mIOU and F1-score reach 81.34% and 89.62%, respectively, which are better than the metrics of the control model.

## 1. Introduction

The range of oil spill pollution is wide and difficult to clean up; therefore, early detection of the oil spill area is of great significance to marine ecology, along with the subsequent cleanup work. Synthetic Aperture Radar (SAR) is a system that actively observes and images the target. Compared with traditional optical remote sensors, SAR is not affected by clouds, rain, and other external environments, and is capable of round-the-clock and all-weather imaging [1,2]. Therefore, it is widely used in military and civilian fields. The use of synthetic aperture radar (SAR), a strong and effective satellite monitoring tool, to monitor sea surface oil spills is also recognized as one of the ideal sensors at home and abroad. When the sea surface is covered by an oil film, the oil film inhibits the formation of short gravity waves and capillary waves at the sea surface and hinders the natural motion of the sea surface, which leads to the weakening of Bragg scattering in the oil spill region. In SAR images, the oil spill area will thus appear as a dark patch, contrasting with the surrounding sea surface area [3,4].

Despite the significant advantages of SAR imaging, whose images are usually characterized by high noise and low contrast, traditional SAR image processing methods, including thresholding-based, morphological processing, and traditional machine learning algorithms, are often ineffective when confronted with high-noise and low-contrast SAR images [5]. These methods usually rely on pre-set parameters and feature extraction techniques, which are difficult to adapt to the complex and changing environment. In recent years, with the rapid development of artificial intelligence technology, deep learning techniques have been applied to sea surface oil spill segmentation using SAR images.

Long et al. [6] proposed Fully Convolutional Networks (FCNs) for the semantic segmentation of images based on anti-convolution and jump connections in 2015. In the same year, Ronneberger et al. [7] proposed a U-Net network based on FCN, which still outperforms other networks under the condition of using less image data for training and ensures that the size of the input image remains the same as the output image. Based on this advantage, the U-Net was applied to the detection of a marine oil spill from SAR images [8,9]. In 2016, Xu et al. [10] put two independent artificial neural networks (ANNs) together to detect a marine oil spill from SAR images sequentially. The first ANN, a backpropagation neural network (BPNN), was used to segment the SAR images to identify black spots caused by oil spills or oil-like objects. Based on the extracted statistical features, the second BPNN is driven to distinguish between oil spills and similar oil spills. In 2017, Song et al. [11] combined multiple fully polarized SAR feature data with an optimized wavelet neural network classifier (WNN) to validate the effectiveness of the proposed method by using two sets of fully polarized RADARSAT-2 SAR data, and the experimental results proved the method’s effectiveness and applicability to marine oil spills. The experimental results prove that the method is effective and applicable to marine oil spill classification. In 2018, Chen et al. [12] developed the DeepLabV3+ network for semantic segmentation. This network introduces a unique encoding–decoding structure to better capture spatial information and boundary details; the encoder part extracts features through multi-scale context aggregation, while the decoder part recovers the spatial resolution. This encoding–decoding structure has also been widely used by researchers in new oil spill detection models. In 2019, Krestenitis et al. [13] combined U-Net, LinkNet, DeepLabV3+, and other mainstream segmentation models’ backbone networks, which were replaced and applied to SAR image sea surface oil spill detection, and the results showed that DeepLabV3+ had the best comprehensive performance. In 2020, Zeng et al. [14] proposed an oil spill convolutional network (OSCNet) with 12 weight layers, which deepens the network by deepening the network depth to better extract features and can learn oil spill detail features from the dataset better than hand-labeled detail features. In 2021, Shaban et al. [15] proposed a deep learning framework that is designed as two parts. The first part is a convolutional neural network (CNN) using a Frost filter, where the final output is then differentiated by whether the number of pixels in the oil spill area accounted for more than 1% or not. The part that is greater than 1% is taken as the input of the second part of the framework, while the part that is less than 1% is directly rounded off. The second part uses a five-layer U-Net and a generalized dice loss function to optimize the inputs, and the oil spill pixel recognition accuracy reached 84%. Optical remote sensing images also play an important role in oil spill detection. Seydi et al. [16] developed a new framework for oil spill detection in optical remote sensing images based on a multiscale multidimensional residual kernel convolutional neural network, which was investigated using a two-dimensional multiscale residual block and applied to a one-dimensional multiscale residual block. Li et al. [17] proposed a deep learning framework based on predicting the probability of semantic spatial position distribution for remote sensing image alignment, which effectively overcame the sensitivity of the traditional methods to radiometric disparity and the problem of long processing time by optimizing the subpixel matching position and determining the semantic spatial probability distribution of the matching template. In 2022, Wang et al. [18] designed the oil spill detection network BO-DRNet using polarization features. Its basic network architecture is based on DeepLabV3+ and the backbone network is replaced with ResNet-18, which makes the network capable of obtaining more complete detailed features of the oil spill while using Bayesian optimization (BO) to adjust the hyperparameters. The experimental results show that the average accuracy of BO-DRNet is 74.69% and the average Dice coefficient is 85.51%. In 2023, to address the problem of oil spill dataset limitation in SAR images, Fan et al. [19] designed a Multi-task Generative Adversarial Network (MTGAN) oil spill detection model, which is capable of distinguishing between oil spills and similar oil spill regions and segmenting the oil spill regions within a single framework. The network only needs to use a small number of oil spill images as a training set for the network, and the experimental results show that the proposed MTGAN oil spill detection framework outperforms other models in oil spill classification and semantic segmentation. In 2024, Li et al. [20] designed an oil spill segmentation network based on U-Net, which mainly consists of a multi-convolutional layer (MCL). The MCL module extracts the basic feature information of the SAR image, and a feature extraction module (FEM) further extracts and fuses different levels of feature maps generated by the U-Net decoder. After the above operations, the network can learn rich global and local contextual information, which improves the segmentation accuracy of the oil spill region. The identification of oil spills in SAR images mainly relies on computer image processing and pattern recognition technology in the aforementioned literature without the utilization of physical information such as the polarization and phase information of SAR images. One of the current state-of-the-art approaches is to combine the physical information of SAR images with neural networks for oil spill detection. Polarized SAR data provide a rich set of polarization features, enabling distinguishing different types of sea surface phenomena [21,22].

The unique imaging mechanism of SAR means that SAR images are characterized by high noise and low contrast; therefore, current methods are not fully applicable to the field of SAR image segmentation. To address the above problems, this paper first utilizes the original DeepLabV3+ model for sea surface oil spill detection using SAR images. The backbone network of this model is Xception; however, experimental results show that the detection performance of Xception–DeepLabV3+ is not good. Therefore, Xception is replaced by a lightweight feature extraction network, MobileNetV2, and the experimental results show that using MobileNetV2 as the backbone network leads to better detection performance than using Xception. Although the overall performance of the MobileNetV2–DeepLabV3+ model is significantly improved, it is still deficient in extracting the details of the oil spill area. For this reason, this paper introduces the spatial and channel Squeeze and Excitation module (scSE) into the MobileNetV2 backbone network and Atrous Spatial Pyramid Pooling (ASPP). Meanwhile, in order to solve the problem of category imbalance between the oil spill region and the sea surface background, the joint loss function of Bce + Dice is adopted. The scSE module improves the model’s focus on the channel and spatial information of the oil spill region by enhancing feature representation, while the joint loss function of Bce + Dice improves the model’s ability to detect the oil spill region by dealing with the category imbalance and optimizing the boundary details, especially in the extraction of the boundary details of the oil spill region, which has a significant effect on the detection of the oil spill area with boundary detail extraction significantly improved.

## 2. SAR Image Sea Surface Oil Spill Detection Model

**Figure 1:**
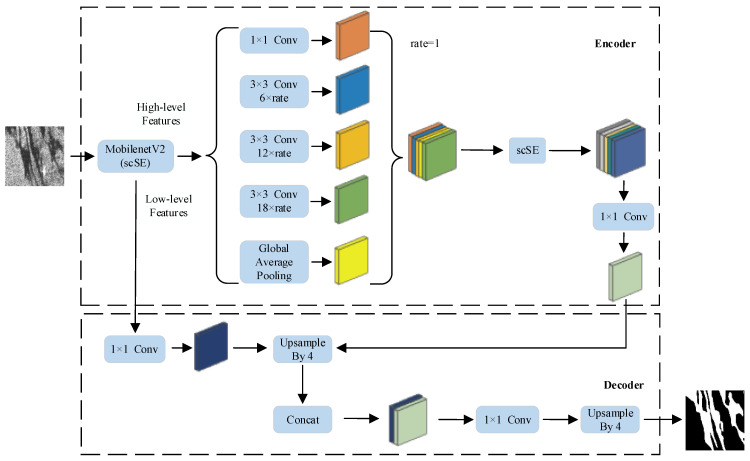
Structure of SAR image oil spill detection model.

An improved DeepLabV3+ model is proposed in this paper for sea surface oil spill detection using SAR images. The model adopts MobileNetV2, incorporating the scSE module as the backbone network, and at the same time adds the scSE module to the ASPP module. The joint loss function (Bce + Dice) is also added to guide model optimization, aiming to improve the detection performance of the model. The model framework is shown in Figure 1, including the Encoder, Decoder, scSE-MobileNetV2, and scSE-ASPP.

### 2.1. Encoder

The encoder in the oil spill detection model consists of a backbone network incorporating the scSE module and the ASPP module, with the main functions of extracting multi-scale oil spill area features and providing enriched features for use by the decoder.

#### 2.1.1. scSE Module

**Figure 2:**
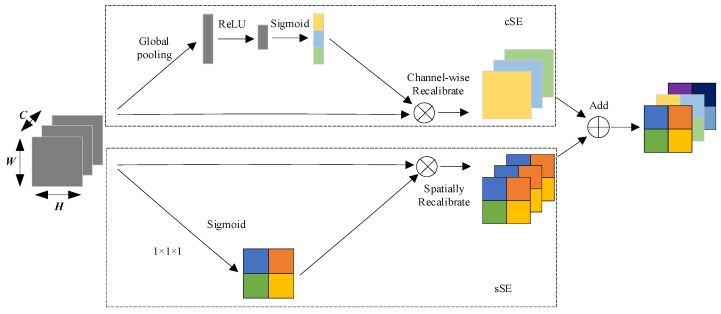
Structure of the scSE module.

To address the problem of the model’s insufficient ability to extract the details of the oil spill area, the scSE module is introduced to reweigh the channels and spatial locations of the oil spill area so that the model can pay more attention to the channel and spatial location information of the oil spill area. The module consists of two sub-modules: channel Squeeze and Excitation (cSE) and spatial Squeeze and Excitation (sSE). The channel and spatial dimensions of the feature map are processed, respectively [23]. The structure of the scSE module is shown in Figure 2.

The role of the cSE module is to recalibrate the channel weights of the feature map through a global channel attention mechanism. The core idea is to obtain the global information of each channel through global average pooling and to adjust the weights of each channel through the fully connected layer.

The input feature maps $F\in R^{C\times H\times W}$ are first globally average pooled to obtain the average value for each channel:

(1) $$z_{c}=\frac{1}{H\times W}\sum_{i=1}^{H}\sum_{j=1}^{W}F_{cij}$$

where $z_{c}$ is the global evaluation pooling value for the cth channel.

The output of global average pooling is passed through two fully connected layers to generate channel weights:

(2) $$s=\sigma (W_{2}\cdot \delta (W_{1}\cdot z))$$

where $W_{1}\in R^{(C/r)\times C}$ and $W_{2}\in R^{C\times (C/r)}$ are the learnable weight matrices and r is the shrinkage rate. δ is the ReLU activation function, defined as δ(x)=max(0,x); σ is the Sigmoid activation function, defined as $\sigma (x)=1/(1+e^{-x})$; and s is the channel weight.

Recalibrate the feature map by multiplying the resulting channel weights s with the input feature map $F\in R^{C\times H\times W}$ by channel:

(3) $$F_{\mathrm{cSE}}=s_{c}\cdot F_{c}$$

where $F_{\mathrm{cSE}}$ is the feature map of the recalibrated channel.

The main purpose of the sSE module is to recalibrate the spatial location weights of the feature map through the spatial attention mechanism. The core idea is to obtain the weight of each spatial location by 1 × 1 convolution and adjust the weight of the spatial location of the oil spill region by the Sigmoid activation function.

The channels of the input feature map $F\in R^{C\times H\times W}$ are compressed into a single channel, and then the spatial weights are obtained by a sigmoid activation function:

(4) $$v=\sigma ({\mathrm{Conv}}_{1\times 1}(F))$$

The spatial weights are multiplied with the input feature map to obtain the recalibrated spatial feature map:

(5) $$F_{\mathrm{sSE}}=v\cdot F$$

Finally, the results of the two parts of cSE and sSE are summed in parallel to obtain the feature map with recalibrated channels and spatial weights:

(6) $$F_{\mathrm{scSE}}=F_{\mathrm{cSE}}+F_{\mathrm{sSE}}$$

#### 2.1.2. scSE–MobileNetV2

MobileNetV2, incorporating the scSE module, is used as the backbone network in the encoder section in order to focus the model on the channel and spatial information of the oil spill area at the initial feature extraction stage. MobileNetV2 ensures a robust extraction capability while maintaining computational efficiency, drastically reducing the parameters through the use of an inverted residual structure, a linear bottleneck, and depth-separable convolution [24]. This lightweight design is not only suitable for resource-constrained environments but also effectively copes with the high noise and low contrast problems prevalent in oil spill SAR images.

**Figure 3:**
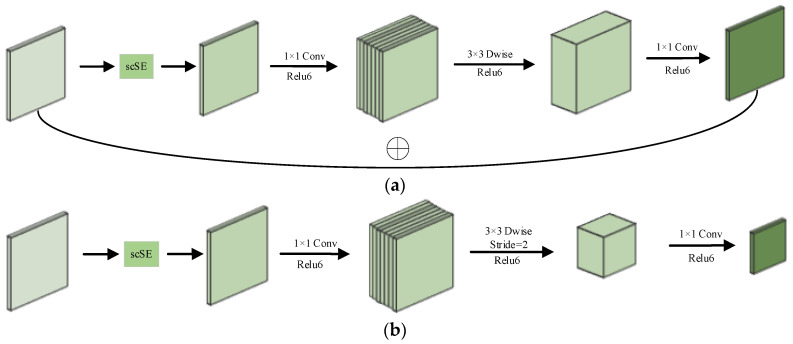
Structure of scSE–MobileNetV2: (**a**) Inverted residual block with a stride of 1; (**b**) Inverted residual block with a stride of 2.

The location of the scSE module insertion in the MobileNetV2 network is shown in Figure 3. Inserting the scSE module after the input of each inverted residual block and before the 1 × 1 convolution can strengthen the features of the oil spill region and suppress the redundant features before the convolution, so as to prevent the original feature information from being mixed and compressed due to the subsequent convolution operation, which weakens the role of the scSE module. For the inverted residual block with a step size of 1, after the input image is recalibrated with channels and spatial weights by the scSE module, the number of channels is first expanded by 1 × 1 convolution, then feature extraction is performed by 3 × 3 depth convolution, and finally, the number of channels is compressed by 1 × 1 convolution. After the final 1 × 1 convolution, the feature map is output directly without using the activation function, which is the linear bottleneck. The main reason for using ReLU6 is to ensure numerical stability and prevent gradient explosion. When the stride is 1 and the number of input and output channels are equal, a residual connection is used to add the input directly to the output. This connection helps to alleviate the gradient vanishing problem and improve the training effect. When the stride is 2, 3 × 3 deep convolution is performed independently for each channel, and the input feature map size becomes one-half of the original. Downsampling is achieved by the above operation, which preserves the local features of the feature map; however, because the input and output feature maps are different sizes, they cannot be directly added, so they are directly output.

#### 2.1.3. scSE–ASPP

After the input image is subjected to feature extraction by the backbone network, the shallow features are fed into the decoder and the deep features are fed into the Airspace Pyramid Pooling (ASPP) module. The ASPP module captures multi-scale feature information by using airspace convolutional layers with different dilation rates (including 1 × 1 convolution, 3 × 3 convolution, and dilation rates of 6, 12, and 18, respectively) and a globally averaged pooling layer. The capture of multi-scale features enables better understanding of the contextual information in the oil spill image, and these features are spliced together and then subjected to further convolutional operations to generate a feature map with rich contextual information. However, these features are interfered with by noise or irrelevant information in the original model, and this interference affects the expressive ability of the features and reduces the detection accuracy of the model for the oil spill area.

**Figure 4:**
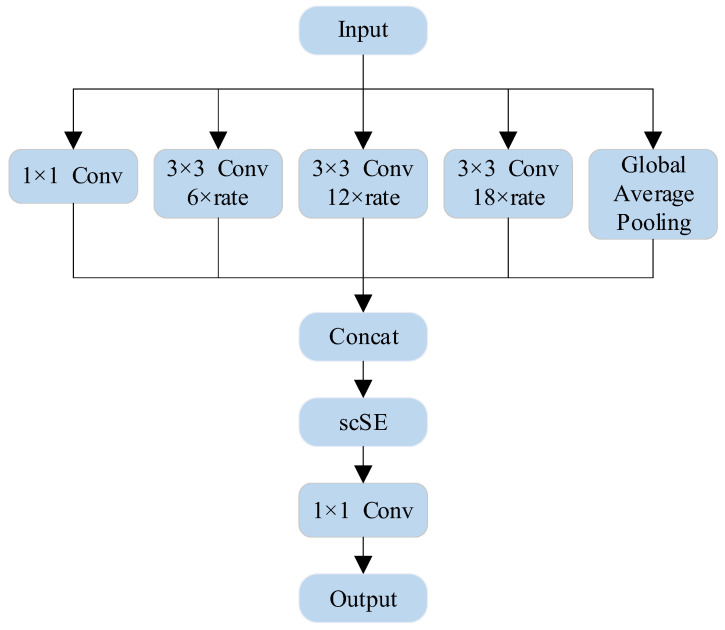
Structure of scSE–ASPP.

As shown in Figure 4, in order to further improve the expression ability of the features of the oil spill area, the scSE module is added after feature splicing and before the channel reorganization of the ASPP module, which can adaptively adjust the importance of each location and channel in the feature map by combining the spatial attention and channel attention mechanisms. This can significantly enhance the model’s attention to the oil spill area, improve the accuracy of feature expression, and ultimately improve the overall detection accuracy.

### 2.2. Decoder

In the decoder section, the model first reduces the size of the feature map output from the encoder to the same size as the shallow features through a 4-fold upsampling operation in order to fuse the shallow features extracted from the backbone network with the deeper features output from the encoder. Then, the number of channels is adjusted by 1 × 1 convolution and the sizes of the fused feature map and the original input image are again made consistent by a 4-fold upsampling operation.

### 2.3. Joint Loss Function

**Figure 5:**
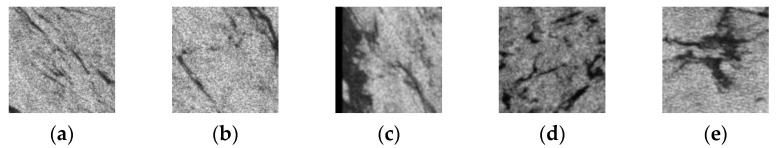
Oil spill SAR images from the ALOS satellite: (**a**) with filename 10777_sat in dataset; (**b**) with filename 10794_sat; (**c**) with filename 11148_sat; (**d**) with filename 11064_sat; (**e**) with filename 11168_sat.

The five SAR images presented in Figure 5 are all from the ALOS satellite and are all pre-processed 256 × 256-pixel images. From Figure 5, the last three images visually appear slightly blurrier than the first two, due to the difference in exaction time and location of the SAR imaging. The oil spill SAR image dataset has the problems of unbalanced data categories, low contrast, and complex boundaries, which lead to the model’s insufficient capture of the detailed features of the oil spill. Therefore, this paper introduces the Bce + Dice joint loss function, which synthesizes the advantages of the Bce loss and Dice loss functions to provide stable classification performance, adapted to the unbalanced data, and strong sensitivity to small targets and complex region shapes.

The joint loss function is defined as follows:

(7) $$L=\alpha L_{\mathrm{Bce}}+\beta L_{\mathrm{Dice}}$$

where $L_{\mathrm{Bce}}$ is the Binary Cross-Entropy loss function, $L_{\mathrm{Dice}}$ is the Dice coefficient loss function; and α, β are weight coefficients. In this study, we use a combination of weight coefficients with α=0.8, β=0.2.

The Bce loss function can effectively distinguish between oil spill regions and non-oil spill regions, providing a clear classification boundary. The oil spill region usually only accounts for a small part of the image. At the same time, the loss function can better deal with the problem of the imbalance of data categories. In this paper, we adopt Binary Cross-Entropy loss as the basic loss function, defined as follows:

(8) $$L_{\mathrm{Bce}}=-\frac{1}{N}\sum_{i=1}^{N}\left[y_{i}\log({\hat{y}}_{i})+(1-y_{i})\log(1-{\hat{y}}_{i})\right]$$

where $y_{i}$ is the true label value (0 or 1) of the ith pixel and ${\hat{y}}_{i}$ is the predicted probability of the ith pixel.

The Dice loss function focuses on the shape and coherence of the oil spill area, which helps to improve the segmentation accuracy, especially for complex oil spill areas. The introduction of the square term makes the loss function more sensitive to larger errors, which improves the robustness of the model [25]. Dice coefficient loss is defined as follows:

(9) $$L_{\mathrm{Dice}}=1-\frac{2\sum_{i=1}^{N}y_{i}{\hat{y}}_{i}}{\sum_{i=1}^{N}\left(y_{i}^{2}+{\hat{y}}_{i}^{2}\right)}$$

## 3. Experimentation and Analysis

### 3.1. Experiment Configuration and Hyperparameter Setting

The oil spill dataset used in this paper is the SOS Oil Spill Detection dataset (Deep-SAR Oil Spill (SOS) dataset) from the team of Qiqi Zhu of China University of Geosciences. The dataset contains the oil spill area in the Gulf of Mexico and the oil spill area in the Persian Gulf, which were acquired from the ALOS and Sentinel-1A satellites, respectively. Among them, the PALSAR dataset is sourced from an explosion that occurred on the Deepwater Horizon drilling platform in the Gulf of Mexico in 2010, which resulted in a massive oil spill. The spill extended approximately 160 km in length and reached a maximum width of around 72 km. The longitude range is 87.056° W–91.371° W and the latitude range is 25.719° N–29.723° N. PALSAR images of the Gulf of Mexico region were acquired between May 2010 and August 2010. PALSAR is a SAR sensor carried by the ALOS satellite and operates in the L-band with HH polarization, enabling cloud-free and day-and-night land observations. The raw data are PALSAR data products (level 1.5), pre-processed by multi-visual processing and a map projection operation, and the observation mode is a polarization method with a pixel spacing of 12.5 m. The oil spill can be clearly shown in the HH polarization image. Therefore, the HH polarization of PALSAR is utilized as the raw data for oil spill detection. The Sentinel-1A satellite is equipped with a C-band SAR sensor with all-weather imaging capability, and a Level-1 Interferometric Wide Swath GRD product was used in this study. The mode image width is 250 km with a spatial resolution of 5 m × 20 m (monovision). The angle of incidence is about 29.1°∼46°. The longitude range is 46.893° E–52.812° E and the latitude range is 25.505° N–48.995° N. The polarization modes are VV and VH. The VV polarization of Sentinel 1A is used as the raw data for oil spill detection. Then, pre-processing operations such as scattering filtering, radiometric calibration, and terrain correction are performed. In this study, we used only the backscattered intensity information of the SAR images. It should be noted that low to moderate wind speeds are recommended as the ideal conditions for detecting oil spills on the ocean surface. Under high wind speeds, oil spills begin to spread and can hardly be observed.

The 21 original SAR images were segmented into 6456 oil spill images 416 × 416 pixels large. Then, labels were produced by manual interpretation. GIS experts sampled the relevant sub-areas. In order to eliminate the imbalance between the number of oil spill samples and the number of background samples, samples with a smaller percentage of oil spill areas were screened. Finally, an oil spill monitoring dataset of 8070 tags with a data size of 256 × 256 pixels was obtained from two satellite platforms. The dataset was divided into training and test sets according to the ratio of 8:2. A total of 3101 images from the Mexican oil spill area were used for training and 776 images were used for testing. A total of 3354 images from the Persian Gulf oil spill area were used for training and 839 images were used for testing [26].

To ensure the fairness of the experiments and the reproducibility of the results, all models were trained and tested under the same conditions. All models were trained and tested on an NVIDIA GeForce RTX4060 GPU (Santa Clara, CA, USA) (with 8G of video memory), with a win11 professional operating system, based on the pytorch framework, python version 3.8 and CUDA version 11.5, an initial learning rate set to 0.001, a batch size of 15, and 80 cycles of iterations using the Adam optimizer [27]. We chose the default 0.5 as the threshold, a prediction probability below the set threshold of 0.5 is judged as background, and a prediction probability above the threshold of 0.5 is judged as the oil spill target.

### 3.2. Evaluation Metrics

In this paper, the mIOU (Mean Intersection and Merger Ratio), F1-score, Precision, and Recall are used as the evaluation metrics of the model. True Positive (TP) indicates that the actual category of the sample is positive and the model correctly recognizes it as positive. False Negative (FN) indicates that the actual category of the sample is positive, but the model incorrectly recognizes it as negative. False Positive (FP) indicates that the actual category of the sample is a negative category, but the model incorrectly recognizes it as a positive category. True Negative (TN) indicates that the actual category of the sample is a negative category and the model correctly identifies it as a negative category.

The mIOU measures the extent to which the model predictions overlap with the true labels, and is the average of all category IOUs (intersection and concurrency ratios), calculated as follows:

(10) $$\mathrm{mIOU}=\frac{1}{k+1}\sum_{i=0}^{k}\frac{TP}{TP+FN+FP}$$

The F1-score is the reconciled average of the model’s Precision and Recall and is calculated as follows:

(11) $$\text{F1-score}=2\times \frac{Precision\times Recall}{Precision+Recall}$$

Precision denotes the proportion of the sample that is predicted to be in the positive category and is actually in the positive category, and is calculated by the following formula:

(12) $$\mathrm{Precision}=\frac{TP}{TP+FP}$$

Recall denotes the proportion of samples in the positive category that are predicted to be in the positive category, and is calculated by the following formula:

(13) $$\mathrm{Recall}=\frac{TP}{TP+FN}$$

### 3.3. Results and Analysis

Firstly, the Xception–DeepLabV3+ model and the MobileNetV2–DeepLabV3+ model were compared and analyzed in two oil spill areas. Then, the improved model of this paper was compared and analyzed with other models in two oil spill areas. Finally, ablation experiments were conducted to demonstrate the positive gain of the improvements in each part.

#### 3.3.1. Comparative Experiments on Different Backbone Networks

Two DeepLabV3+ models with different backbone networks are quantitatively and qualitatively analyzed and compared based on evaluation metrics, model parameters, and visualization results.

Table 1 shows a comparison of evaluation metrics for DeepLabV3+ models with different backbone networks in the Gulf of Mexico oil spill area. Table 2 shows a comparison of evaluation metrics for DeepLabV3+ models with different backbone networks in the Persian Gulf oil spill area. From Table 1 and Table 2, it can be seen that MobileNetV2–DeepLabV3+ outperforms Xception–DeepLabV3+ in all the metrics in the two oil spill areas. Among them, in the Gulf of Mexico oil spill area, the difference between the mIOU and F1-score is 24.50% and 16.25%, and in the Persian Gulf oil spill area, the difference between the mIOU and F1-score is 34.42% and 24.69%. This is due to the fact that the network structure of Xception is more complex than MobileNetV2, which is easily interfered with by noise when dealing with high-noise SAR images, resulting in unstable feature extraction and degradation of detection accuracy. The effectiveness of MobileNetV2–DeepLabV3+ is verified in terms of evaluation metrics.

Table 3 shows the comparison of DeepLabV3+ model parameters for different backbone networks, where the total number of parameters and the total memory of MobileNetV2–DeepLabV3+ are about one-ninth of those of Xception–DeepLabV3+, and the total floating-point operations are about one-third of those of Xception–DeepLabV3+. The complex network structure of Xception leads to its more demanding computer hardware configuration. The network structure results in higher requirements for computer hardware configuration. The effectiveness of MobileNetV2–DeepLabV3+ is verified in terms of complexity, memory requirements, and computational volume.

**Figure 6:**
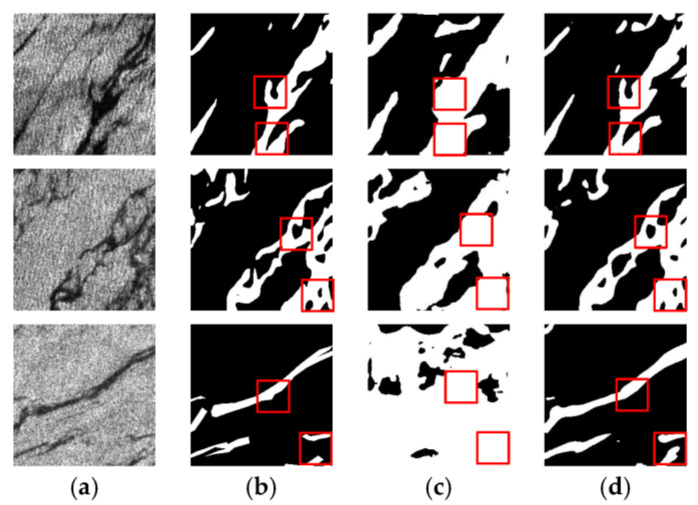
Prediction results of different backbone networks in the Gulf of Mexico oil spill area: (**a**) SAR image; (**b**) Ground truth; (**c**) Xception–DeepLabV3+ model; (**d**) MobileNetV2–DeepLabV3+ model.

**Figure 7:**
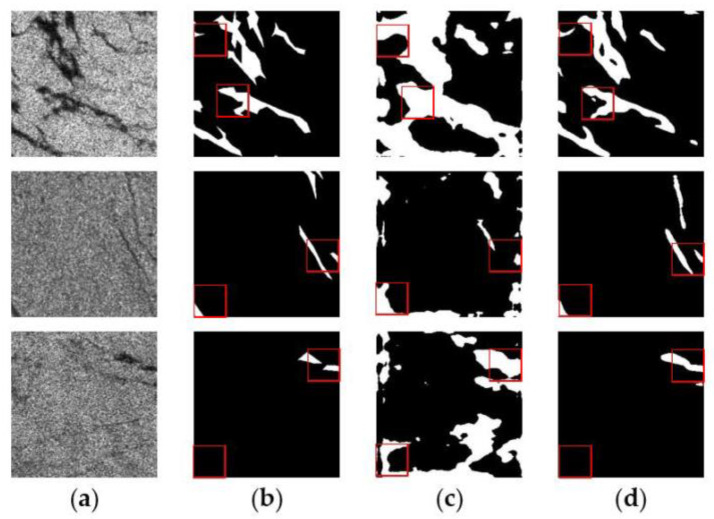
Prediction results of different backbone networks in the Persian Gulf oil spill area: (**a**) SAR image; (**b**) Ground truth; (**c**) Xception–DeepLabV3+ model; (**d**) MobileNetV2–DeepLabV3+ model.

Figure 6 and Figure 7 show the comparison of the prediction results of DeepLabV3+ models with different backbone networks in two oil spill areas. The white color in the images represents the oil spill area and the black color represents the sea surface background. The red boxes in the Figures highlight the detailed parts of the prediction results using different models. It can be seen that in both oil spill areas, Xception–DeepLabV3+ has a lot of misses and misdetections compared to MobileNetV2–DeepLabV3+. In addition, the boundary part is rough, and the presence of white spots is due to noise accumulation. As shown in the third row of results in Figure 6 and the third row of results in Figure 7, Xception–DeepLabV3+ erroneously learns a large amount of noise, which leads to misdetection of the background area of the sea surface as an oil spill area.

Comprehensively considering the metrics, model parameters, and visualization results, it can be concluded that the MobileNetV2–DeepLabV3+ model can provide more efficient feature extraction and noise suppression capabilities in the task of sea surface oil spill detection in SAR images, which is applicable to the characteristics of SAR images with high noise and low contrast, thus exhibiting better detection results. Xception, on the other hand, has a poor detection effect due to the accumulation of noise caused by its complex network structure, which leads to omission, misdetection, and noise patches in the predicted images [28,29].

#### 3.3.2. Comparative Experiments with Different Models

The improved model proposed in this paper is compared with six other models, ABCNet [30], CGNet [31], DFANet [32], LEDNet [33], MANet [34], and UNet [7], in two oil spill regions, respectively. Comparative analysis is achieved in terms of evaluation metrics, model parameters, and visualization results.

Table 4 shows the comparison of the metrics between the improved model of this paper and other segmentation models in the Gulf of Mexico oil spill area, and Table 5 shows the comparison of the metrics between the improved model of this paper and other models in the Persian Gulf oil spill area. In the Gulf of Mexico oil spill area, although the recall of the MANet model is 0.06% higher than that of the improved model of this paper, the improved model of this paper has a higher F1-score, which indicates that the improved model of this paper can maintain a balance between precision and recall. In the Persian Gulf oil spill region, all the metrics of this paper’s improved model are higher than the other models’. The metrics from the two oil spill regions prove the effectiveness of the improved model.

As shown in Table 6, high-parameter, high-memory, and high-computation models (e.g., MANet and ABCNet) are suitable for scenarios requiring high accuracy and complex feature extraction; however, for high-noise SAR images, noise is extracted along with the oil spill features of complex shapes, which results in a decrease in detection accuracy. Low-parameter, low-memory, low-computation models (e.g., CGNet) are suitable for resource-constrained environments and can be used in real-time applications, but are less accurate in the complex task of confronting characteristic oil spill features. The improved model in this paper has the highest of all detection metrics while maintaining lower memory and computation requirements, indicating that the model achieves a good balance between resources and performance.

**Figure 8:**
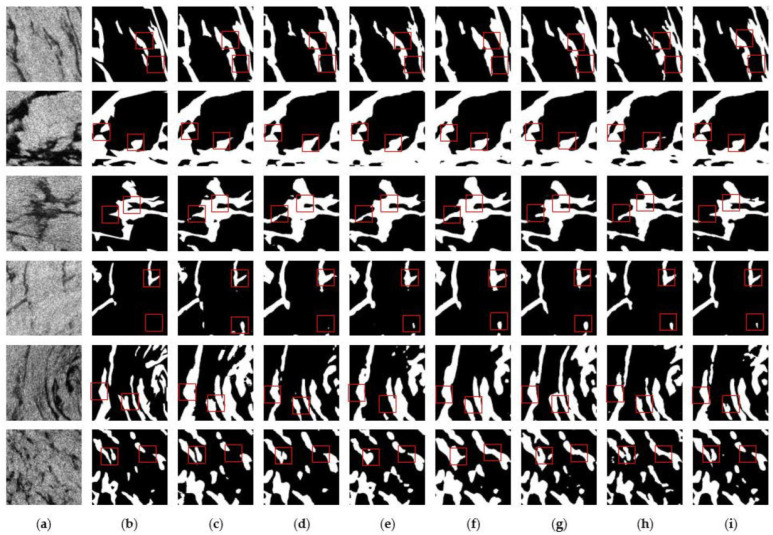
Prediction results of different models in the Gulf of Mexico oil spill area: (**a**) SAR image; (**b**) Ground truth; (**c**) ABCNet; (**d**) CGNet; (**e**) DFANet; (**f**) LEDNet; (**g**) MANet; (**h**) UNet; (**i**) Ours.

**Figure 9:**
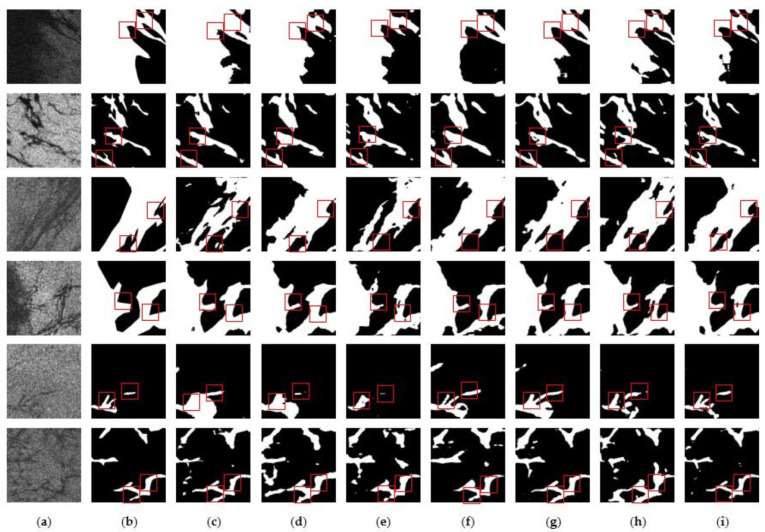
Prediction results of different models in the Persian Gulf oil spill area: (**a**) SAR image; (**b**) Ground truth; (**c**) ABCNet; (**d**) CGNet; (**e**) DFANet; (**f**) LEDNet; (**g**) MANet; (**h**) UNet; (**i**) Ours.

Figure 8 shows the comparison of the prediction results between the control model and the improved model of this paper in the Gulf of Mexico oil spill area, and Figure 9 shows the comparison of the prediction results between the control model and the improved model of this paper in the Persian Gulf oil spill area. The red boxes in the Figures highlight the detailed parts of the prediction results using different models. It can be seen that the improved model of this paper outperforms the other control models in terms of prediction accuracy and detail. As shown in the third row of results in Figure 8 and the fifth row of results in Figure 9, the improved model of this paper more accurately predicts the shape and size of the oil spill area compared with other models due to the introduction of the scSE module and the joint loss function, which makes the model more sensitive to small targets and the shape of the area. It fully proves the superiority of the improved model in this paper in reducing the false detection rate and improving segmentation accuracy.

#### 3.3.3. Ablation Experiments

In order to better evaluate the contribution of each module, ablation experiments are conducted in two oil spill regions separately. MobileNetV2–DeepLabV3+ is used as the benchmark model, the scSE module is added to the backbone network MobileNetV2 and the ASPP of the benchmark model, respectively, and the joint loss function is used to replace the Bce loss function in the benchmark model.

Table 7 shows the comparison of the results of the ablation experiments in the Gulf of Mexico oil spill area. As shown in the results in the second and third rows of Table 7, the mIOU is improved by 1.98% and 2.88% with the addition of the scSE module to ASPP and MobileNetV2, respectively. This indicates that the scSE module makes the model pay more attention to the channel and spatial location information of the oil spill area. When the joint loss function is introduced, the mIOU improves by 1.58%, which proves that the joint loss function can provide stable classification performance, adapt to the unbalanced data, and possess strong sensitivity to complex oil spill shapes to improve the detection accuracy. The combination of the final modules can improve the comprehensive detection performance of the model.

Table 8 shows the comparative results of the ablation experiments in the Persian Gulf oil spill area. After adding the scSE module to MobileNetV2 and ASPP, respectively, the mIOU improves to 78.63% and 79.11%, and the F1-score improves to 88.06% and 88.36%, respectively. These enhancements are related to the ability of the scSE module to improve the integrity of the oil spill target. The results using the joint loss function show a significant improvement in the metrics, proving the effectiveness of the joint loss function.

## 4. Discussion

In this article, our approach relies heavily on a traditional neural network architecture, which performs well in processing standard SAR images (which primarily utilize backscatter intensity information), especially in the binary classification task of detecting oil spills versus the sea surface background. In our study, physical information such as polarization information and phase information has not yet been integrated with neural networks. However, relying only on neural networks for feature extraction is inadequate when dealing with more complex multi-classification scenarios.

One of the current state-of-the-art approaches is to combine the polarization information of SAR with neural networks for oil spill detection. Polarized SAR data provide a rich set of polarization features, enabling distinguishing different types of sea surface phenomena. By utilizing polarization features, oil spills can be more accurately distinguished from other similar objects such as algae, ice floes, and biofilms, resulting in a significant reduction in the false detection rate and improvement of detection accuracy. This is because the polarization information in SAR images is rich in physical features of the target, which can provide rich information about the target’s surface structure, material composition, and the interaction of electromagnetic waves with the target, which are crucial for accurately distinguishing oil spills from other similar objects [35,36]. Hybrid-pol SAR offers a good level of polarimetric information along with shorter satellite revisit times. A shorter revisit time helps detect oil spills at a minor level before they can cause widespread damage [37].

Since our study has not yet integrated physical information such as polarization information, it may be somewhat limited when dealing with complex marine environments. For example, in scenes where multiple similar objects exist, the model may have difficulty in accurately distinguishing oil spills from other objects with similar reflective properties, leading to an increased false detection rate. In addition, the model may have limitations in capturing subtle features and coping with variable ocean conditions due to the failure to fully utilize polarization information.

Based on the above analysis, it provides us with possible future research directions:

Physical information-integrated neural networks will be developed by utilizing physical features such as the polarization and phase information of SAR images. Neural networks integrated with physical information enable them to learn the physical features of oil spills in SAR images, thereby improving the model’s physical consistency, interpretability, and predictive performance. Specifically, we will explore the use of individual polarization channels of SAR images as independent inputs for feature extraction using Convolutional Neural Networks or other sophisticated deep learning architectures. This will help the model take full advantage of the polarization information and enhance its ability to distinguish oil spills from other marine phenomena.

## 5. Conclusions

Aiming at the problems of overfitting and insufficient detail feature capturing in processing oil spill SAR images in the current methods, this paper proposes an improved model based on DeepLabV3+, which uses scSE–MobileNetV2, a backbone network that integrates the attention mechanism in the encoder part and also adds the attention module in the ASPP module to recalibrate the channels of the oil spill region and the spatial weights. In addition, the joint loss function of Bce + Dice is used, which not only performs stably during classification but also adapts to the unbalanced oil spill region and sea surface background. Furthermore, it has higher sensitivity to small targets and complex region shapes, thus improving the detection accuracy. The experimental results show that using MobileNetV2 not only significantly improves the detection accuracy but also reduces the number of computational parameters compared with the DeepLabV3+ model with Xception as the backbone network. In the comparison tests of two oil spill areas in the Gulf of Mexico and the Persian Gulf, the mIOU and F1-score of the improved model reach 80.26% and 88.66% and 81.34% and 89.62%, respectively, which are better than the six comparison models (ABCNet, CGNet, DFANet, LEDNet, MANet, and UNet). Through the optimization and enhancement of key modules, the improved model in this paper not only exhibits higher accuracy and robustness in all performance indexes when dealing with oil spill SAR images with high noise, low contrast, and irregular boundaries but also provides a reliable reference for future oil spill detection research.

## Figures and Tables

**Table 1:** Metrics of different backbone networks in the Gulf of Mexico oil spill area.

Model	mIOU (%)	F1-Score (%)	Precision (%)	Recall (%)
Xception–DeepLabV3+	52.22	69.98	65.62	75.02
MobileNetV2–DeepLabV3+	76.72	86.23	84.58	87.76

**Table 2:** Metrics of different backbone networks in the Persian Gulf oil spill area.

Model	mIOU (%)	F1-Score (%)	Precision (%)	Recall (%)
Xception–DeepLabV3+	43.92	63.05	62.45	63.08
MobileNetV2–DeepLabV3+	78.34	87.74	87.46	88.05

**Table 3:** Model parameters of different backbone networks.

Model	Total Parameters (M)	Total Memory (MB)	Total Flops (G)
Xception–DeepLabV3+	54.71	208.70	19.42
MobileNetV2–DeepLabV3+	5.82	22.18	6.15

**Table 4:** Metrics of different models in the Gulf of Mexico oil spill area.

Model	mIOU (%)	F1-Score (%)	Precision (%)	Recall (%)
ABCNet	78.54	87.67	85.19	90.30
CGNet	78.56	87.62	85.40	89.97
DFANet	78.22	87.20	86.15	88.29
LEDNet	76.49	86.27	83.76	88.94
MANet	78.65	87.76	85.21	90.46
UNet	77.20	86.51	85.28	87.76
Ours	80.26	88.66	87.04	90.40

**Table 5:** Metrics of different models in the Persian Gulf oil spill area.

Model	mIOU (%)	F1-Score (%)	Precision (%)	Recall (%)
ABCNet	79.31	88.52	87.69	89.36
CGNet	80.15	88.83	88.54	89.29
DFANet	79.62	88.55	88.89	88.21
LEDNet	76.62	86.62	86.42	86.85
MANet	79.19	88.29	88.07	88.51
UNet	77.67	87.31	86.99	87.67
Ours	81.34	89.62	89.68	89.56

**Table 6:** Parameters of different models.

Model	Total Parameters (M)	Total Memory (MB)	Total Flops (G)
ABCNet	13.52	51.57	3.58
CGNet	0.49	1.88	0.83
DFANet	2.02	7.68	3.22
LEDNet	2.27	8.66	1.40
MANet	35.86	136.79	18.05
UNet	7.76	29.60	12.74
Ours	5.84	22.28	6.16

**Table 7:** Ablation experiments in the Gulf of Mexico oil spill area.

Baseline	scSE–ASPP	scSE–MoblieNetV2	Joint Loss	mIOU (%)	F1-Score (%)
✓				76.72	86.23
✓	✓			78.70	87.71
✓		✓		79.60	88.07
✓			✓	78.30	87.42
✓	✓	✓	✓	80.26	88.66

**Table 8:** Ablation experiments in the Persian Gulf oil spill area.

Baseline	scSE–ASPP	scSE–MoblieNetV2	Joint Loss	mIOU (%)	F1-Score (%)
✓				78.34	87.74
✓	✓			79.11	88.36
✓		✓		78.63	88.06
✓			✓	79.27	88.42
✓	✓	✓	✓	81.34	89.62

## Data Availability

The dataset used in this study can be found in https://grzy.cug.edu.cn/zhuqiqi.
